# Supplementary material for: Evaluation of Bacterial Diversity and Evolutionary Dynamics of Gut Bifidobacterium longum Isolates Obtained from Older Individuals in Hubei Province, China
Source: Microbiol Spectr. 2022 Jan 19;10(1):e01442-21. doi: 10.1128/spectrum.01442-21 (PMC8768838; doi:10.1128/spectrum.01442-21)
Supplement: SUPPLEMENTAL FILE 4 — Supplemental material. Download SPECTRUM01442-21_Supp_4_seq12.pdf, PDF file, 1.1 MB [file spectrum01442-21_supp_4_seq12.pdf]

Figure S1: Location of the sampling sites and Rarefaction curves.

(A) Sampling sites XY and ES refer to Xiangyang city (108.93°-108.98°E, 30.28°-30.31°N) and Enshi city (111.85°E, 32.04°N), Hubei Province, P.R. China, respectively. (B) Hubei province in China. Rarefaction curves of the Shannon index (C) and observed species index (D). The rarefaction curves can be used to evaluate the adequacy of sequencing for each sample. The sequencing depth was 36,010. ES and XY refer to Enshi city and Xiangyang city in Hubei Province, China, respectively.

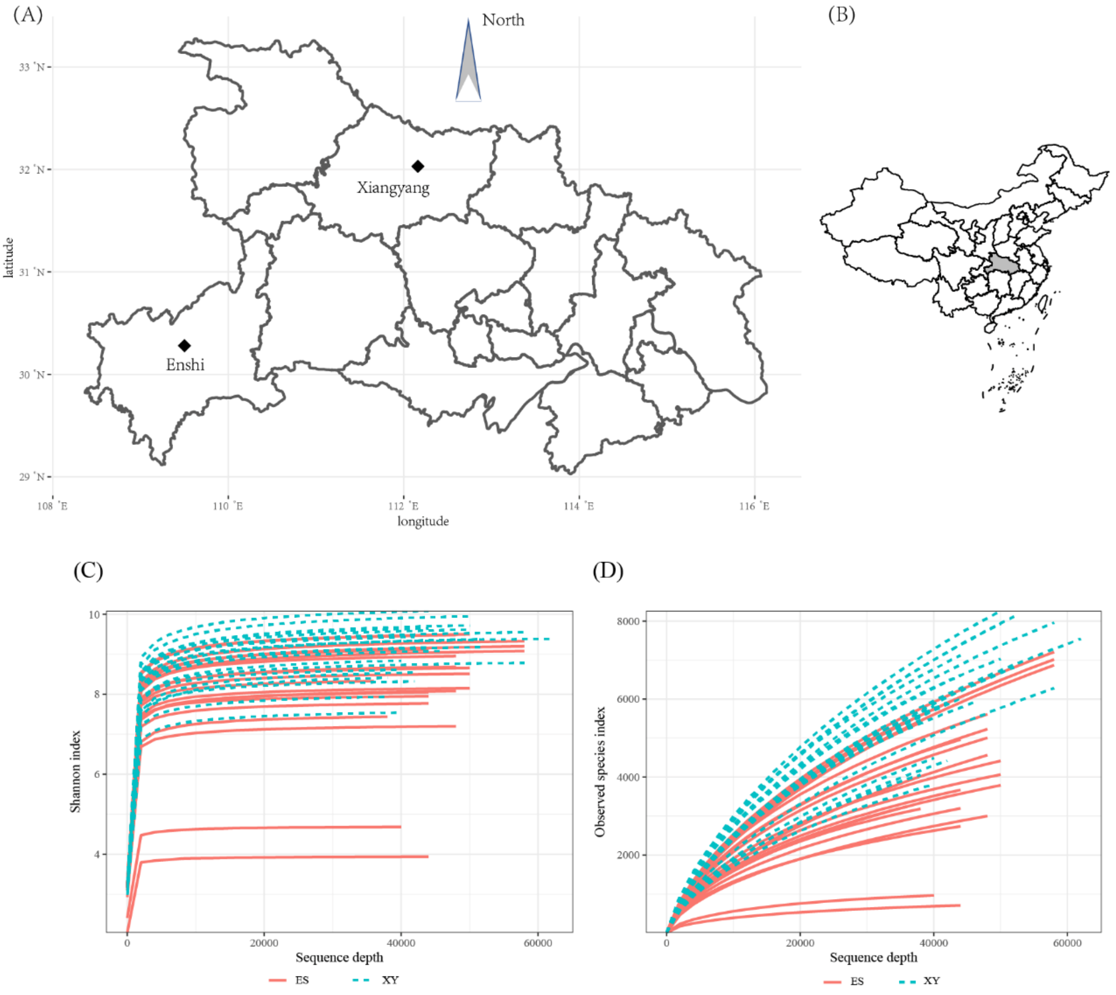

Figure S2: Heatmap indicating for the percent ANI (A), GC content (B), and genomic length (C) of the 407 genomes used for MLST analysis.

ANI was calculated using pyANI v.0.3.0. The gradient from gray (lower ANI) to red (higher ANI) indicates the ANI values. The thick line in the boxplot indicates the median, and the bottom and top edges of the box indicate the 25th and 75th percentiles, respectively.

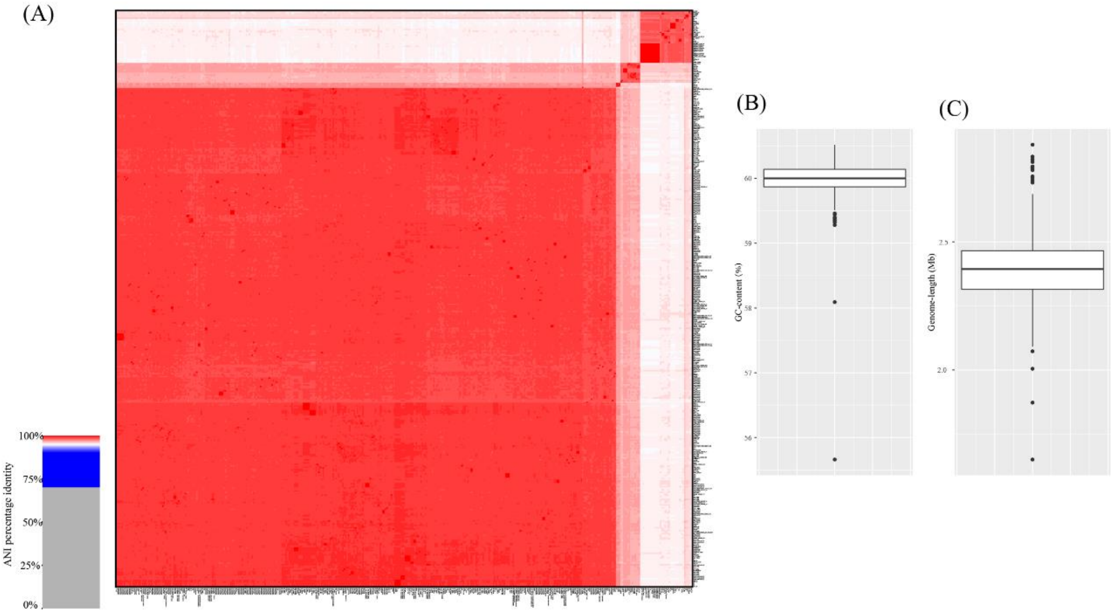

Figure S3: Minimum spanning tree analysis of 437 *B. longum* isolates based on the allelic profiles of six MLST loci according to the region of isolation.

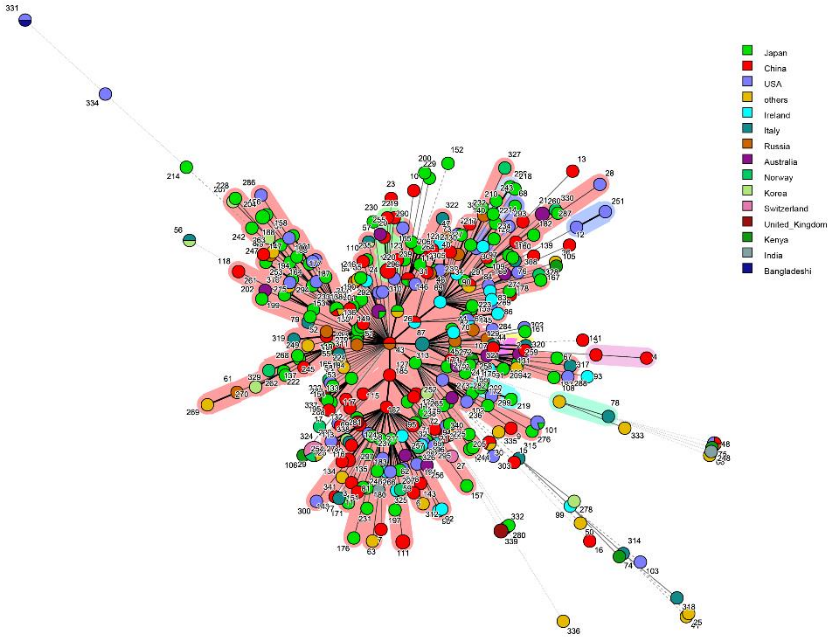

Figure S4: Split network analysis for each of the six MLST loci using SplitsTree v4.

Split network for *clpc* (A), *purF* (B), *fusA* (C), *rplB* (D), *ileS* (E), *rpoB* (F) of the 341 STs, and concatenated sequence of the six MLST loci of STs from Xiangyang (G) and Enshi city (H).

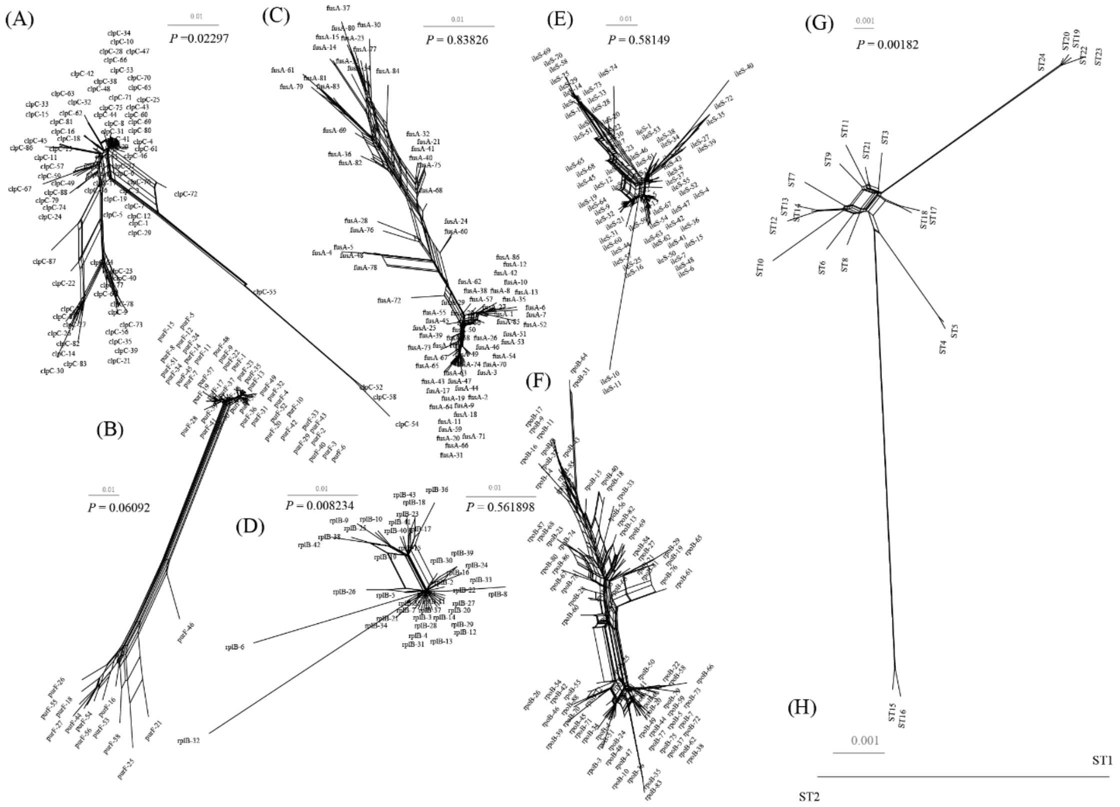

Figure S5: Analysis of recombination in *B. longum* isolates based on the 6 MLST loci (3177 bp) using Gubbins.

The NJ tree on the left was constructed using mega vX with 1000 bootstrap replicates. Blue represents recombination blocks identified at a terminal node, whereas red indicates blocks identified at internal nodes and thus inherited by multiple samples.

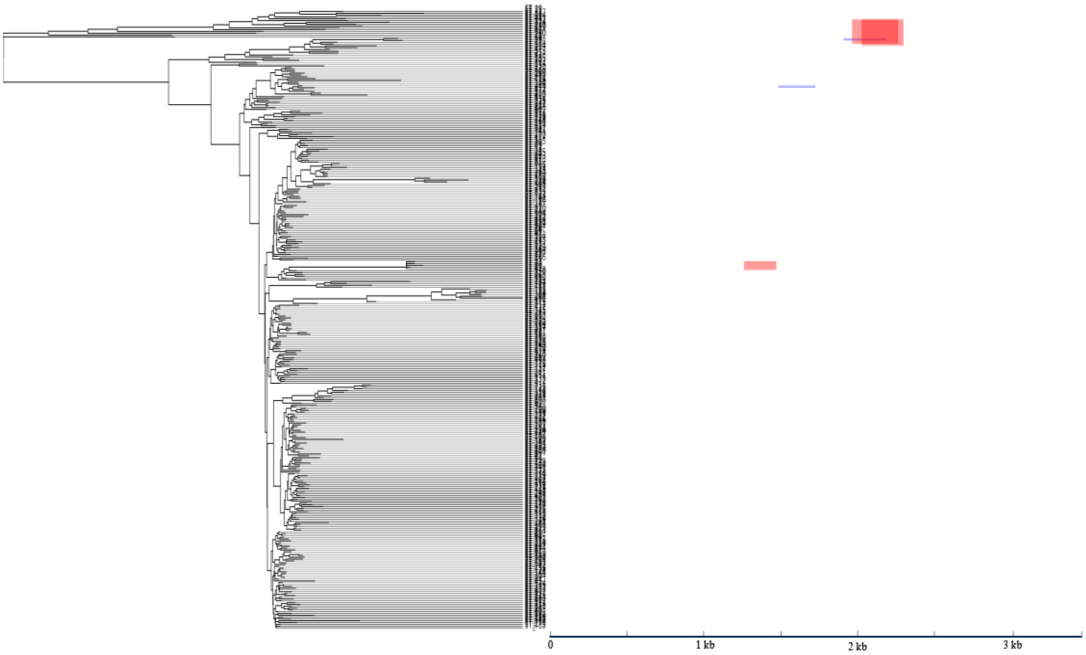

Figure S6: Phylogenetic tree of 407 *B. longum* genomes using the concatenated sequences of 253 single-copy genes.

The single-copy genes were identified and concatenated, and the concatenated sequences were used to construct a maximum likelihood tree using orthofinder v2.5.2.

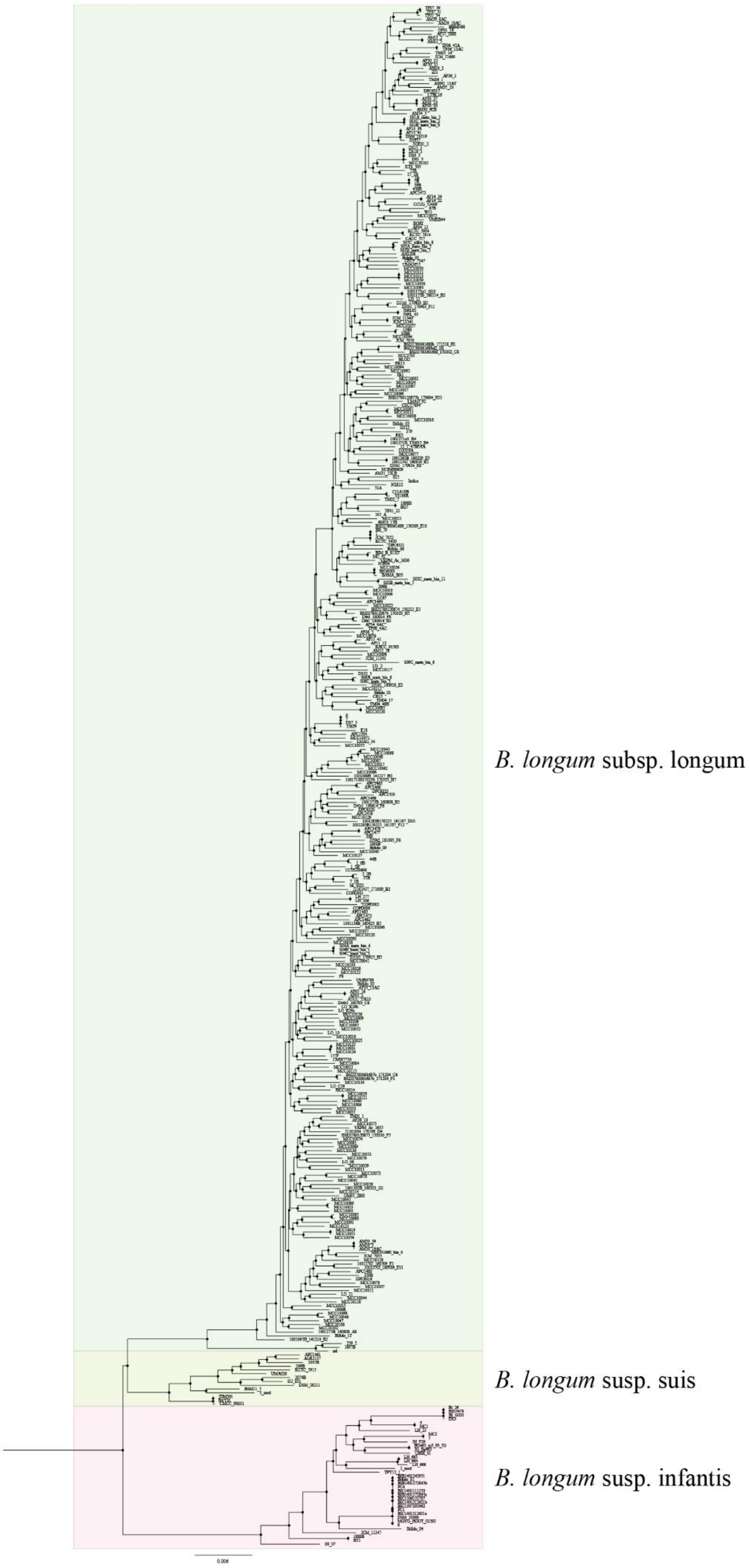
B. longum subsp. longum
B. longum susp. suis
B. longum susp. infantis
0.006
